# Material Extrusion Based Fabrication of Surgical Implant Template and Accuracy Analysis

**DOI:** 10.3390/ma15051738

**Published:** 2022-02-25

**Authors:** Chengyu Zhang, Yanping Yuan, Jimin Chen

**Affiliations:** 1 Beijing Engineering Research Center of 3D Printing for Digital Medical Health, Beijing University of Technology, Beijing 100124, China; zhang.chengyu2009@163.com (C.Z.); yuanyp@bjut.edu.cn (Y.Y.); 2 Beijing International Science and Technology Cooperation Base of 3D Printing for Digital Medical Health, Beijing University of Technology, Beijing 100124, China; 3 Institute of Laser Engineering, Faculty of Materials and Manufacturing, Beijing University of Technology, Beijing 100124, China

**Keywords:** 3D printing, fused deposition modeling, implant template, accuracy analysis, CBCT

## Abstract

An implant template with great precision is significantly critical for clinical application. Currently, the application of an immediate implant remains limited by the deviations between the planned and actual achieved positions and long periods required for preparation of implant templates. Material Extrusion (MEX), as one kind of 3D printing method, is well known for its low cost and easy operation. However, the accuracy of the implant template printed by MEX has not been fully researched. To investigate the accuracy and feasibility of in vitro computer-guided surgery assisted with a MEX printed template, unidentified plaster samples missing a maxillary molar are digitalized. Mimics software (Materialise, Leuven, Belgium) is used for preoperative design. Surgical templates are fabricated by a MEX 3D printer (Lingtong III, Beijing SHINO, Beijing, China). Postoperative CBCT data are obtained after surgical template placement. The differences in positions of X, Y, Z, and dXYZ as well as angulations between the placed and the designed template are measured on labiolingual and mesiodistal planes. The deviations of the planned and the actual outcome in each dimension are observed and analyzed. Data from different samples indicate that the mean deviation of the angle measures approximately 3.640°. For position deviation, the maximum deviation is found in the z-direction and the mean deviation is about 0.365 ± 0.136 mm. The mean deviation of space Euclidean distance dXYZ is approximately 0.537 ± 0.123 mm. Implant templates fabricated by MEX present a relatively high accuracy for tooth-supported guide implantation.

## 1. Introduction

The application of digital technology in the field of dental implantation shows the oral medicine industry has entered a new digital era [1]. Computer-aided design and computer-aided manufacturing (CAD–CAM) technology were broadly applied in the field of dentistry [2,3]. In implant surgery, additive manufacturing or three-dimensional printing (3DP) with the use of stereolithographic technology is widely implemented. The advent of 3D printed templates not only reduces the requirements of clinical experience for implant surgery, but also greatly improves the efficiency [4,5,6]. Imaging research provides the basis for producing implant plans. The emergence of cone-beam computed tomography (CBCT) has solved the shortcomings of apical and curved tomography and is suitable for preimplantation planning of dental implants [7]. Moreover, CBCT data can be used to evaluate the accuracy of 3D printed templates [2]. By comparing the postoperative CBCT scan information with the preoperative planning model, the position and angle deviations can be measured and the accuracy of the implant template can be evaluated [8,9]. The combination of 3D printing for model fabrication and CBCT for data acquisition has rendered it more accurate and convenient for dentists to achieve the objective of immediate implantation.

Material extrusion (MEX) 3D printing technology, also known as Fused Deposition Modeling (FDM) or Fused Filament Fabrication (FFF), is a 3D printing technology that emerged after vat polymerization [10]. MEX has become a widely used 3D printing technology due to its small size and convenience in operation [11]. The advantages of MEX technology are as follows: (1) Almost no pollution and less environmental requirements; (2) Parts can be formed immediately; (3) Low cost for the general public; (4) Users can choose a variety of materials according to their needs, including ABS (Acrylonitrile Butadiene Styrene), PLA (Polylactic Acid), PPSF (Polyphenylsulfone), PC (Polycarbonate), nylon, engineering plastics, et.al. [12]; (5) Simple post-processing steps. Parts can be applied after simple post-processing.

PLA has been widely applied in the medical implantation field owing to its excellent biocompatibility and preferable mechanical properties. It is a biomaterial approved by the American Food and Drug Administration (FDA) and can be biodegraded in vivo [13]. PLA-based templates are preferable over other metal materials as their hydrophobic properties prevent the rapid dissolution of gentamicin [14]. In dentistry, PLA-based materials represents the most used equipment as a fixation-device such as, for example, screws, pins and arrows in regenerative surgeries [15]; fixation for tissue and alignment of bone fragments [16]. In the study of PLA 3D printing process and mechanical properties, research has shown that the 3D printed PLA parts exhibit preferable impact resistance, tensile strength and compressive strength [17]. For example, Afonso, et.al [18] evaluated the influence of printing parameters on the impact resistance of the printed parts. They found that the printing orientation represented the major parameter affecting impact resistance. Kumar, et.al. [19] discovered that the extrusion temperature was the parameter of greatest influence on the mechanical strength, as it changed the thermal/rheological characteristics of the PLA filament. In a study to reveal the influence of porosity, crystallinity and interlayer adhesion on the tensile strength of the 3D printed PLA, Windheim, et.al. [20] demonstrated that PLA samples annealed below cold crystallization temperature (Tcc) did not significantly enhance the porosity or crystallinity. However, the tensile strength increased.

Currently, however, the combination of 3D printing technology and guide template manufacturing technology always involves curable resin and light-based 3D printing methods. Templates are produced by a dedicated 3D printing center, which always typically requires seven or more business days. Moreover, although MEX technology has been successfully applied to computer-aided implant surgery shown by earlier research [21,22], there are few studies that focus on the precision of the MEX-based surgery guide in placement position [23]. In view of the above problems, a method to fabricate surgical guides by MEX and to predict the accuracy in vitro is proposed in this study.

## 2. Materials and Methods

### 2.1. Data Acquisition

A total of 20 maxillary cast models made of resin with a missing molar are chosen to simulate the clinical situation in this research. Simulated bone is placed inside the edentulous area for evaluation of the class II bone quality. A cone-beam computed tomography (CBCT, Newtom VGI) data set of casts from 20 unidentified samples is used as base models. The scan settings are set to 6 cm × 6 cm, 75 μm voxel size to capture the optimal dose algorithm and obtain the best image. Voltage and current are 110 kV and 0.55 mA, respectively. Image acquisition time is 5.4 s. The scanned samples using the same settings are obtained in Digital Medical Imaging and Communication (DICOM) file format [24]. All selected subjects were informed for inclusion of this research previously. The research was performed on the basis of the Helsinki Declaration of 1975 as revised in 2013, also obtaining approval from the ethical committee (BJUTIRB-2017-113).

### 2.2. Implant Planning and Guide Design

**Figure 1:**
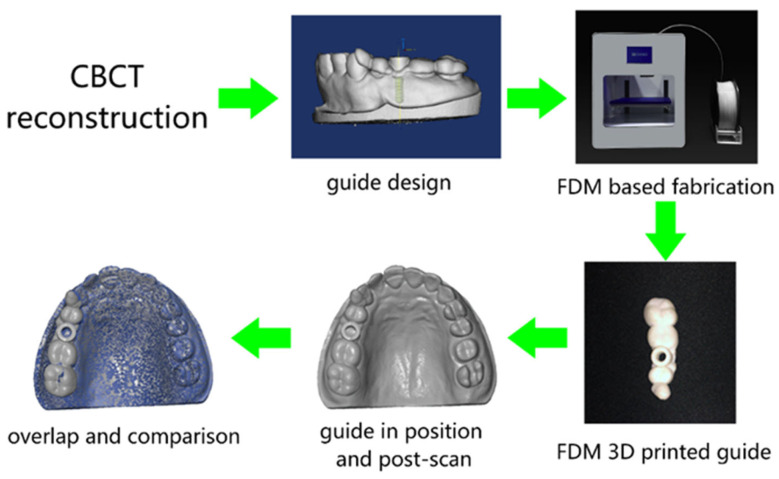
Schematic review of the overall workflow.

The general workflow is shown in Figure 1. CBCT scanned data is imported to software Mimics and a digital model of missing teeth is reconstructed. On the basis of the quality and quantity of alveolar bone indicated by CBCT in the edentulous area, a bone-level implant of Straumann (Straumann AG, Basel, Switzerland) with a length and diameter of, respectively, 10 mm and 4.1 mm is applied. After crown selection, shape and position adjustment at the center of adjacent teeth’ occlusal surface, an optimized position of the implant placement is obtained and the surgical templates supported by adjacent teeth are designed.

### 2.3. Guide Template Fabrication

The designed 3D template models are then imported to the slicing software Dental 100 (Beijing SHINO, Beijing, China) for fabrication in the MEX 3D printer (Lingtong III, Beijing SHINO, Beijing, China). To fulfill the surface quality requirement of the surgical template, the nozzle size of the printer is specifically selected as 300 μm. In addition, given the fact that the slicing parameters are also of great importance, the specific parameters in the slicing software are selected as “FINE” mode. Specific print parameters are shown in Table 1. After slicing, a G-code file is generated and transferred to the MEX printer. The MEX printer used in this research, as shown in Figure 1, is a Core-XY structured mini-printer with a fabrication volume of 180 mm × 160 mm × 150 mm. PLA filament (purchased from Esun3D, Shenzhen, China) material is used for the fabrication of templates due to its non-toxic properties [25]. After the templates have been fabricated, post-processing including support material removal and simple polishing is undertaken. The templates are then placed in the maxillary cast model waiting for a secondary optical scan for comparison of the deviations. The rescanned parts of maxillary casts and printed implant templates are together reconstructed into 3D models for further analysis.

### 2.4. Accuracy Measurements

**Figure 2:**
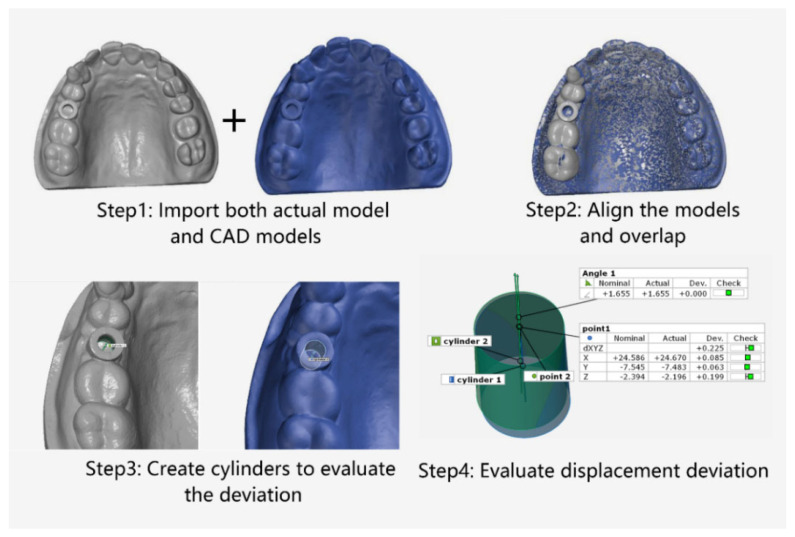
Schematic of the preparation for comparison of deviation based on MEX printed implant templates.

The software GOM Inspect (GOM, ZEISS Group, Braunschweig, Germany) was employed for model comparison. The designed model was set as the nominal, and MEX printed template was used as the actual model. The digital workflow of the evaluation process was mainly divided into four stages [5], as shown in Figure 2: Stage 1: Import the design and actual models. Stage 2: Overlap the designed and actual template models based on the 3-point alignment method in GOM Inspect. Stage 3: Create cylindrical evaluation geometries based on the corresponding implant template sleeves. Stage 4: Assess the deviations between the two implant templates in dimensions (X, Y, Z and dXYZ) and angles through the cylinder. Adjust the coordinate system to the implant template in the following order: X-plane in the vestibular–oral direction, Y-plane in the mid-distal direction and Z-plane in the cranio–caudal direction. Two models were then overlapped on the basis of the cast model (transition deviation of 0.005 mm). With the help of the GOM software, the internal geometry of the implant cannula was selected as a virtual cylinder. The height of the cylinder is limited to the size of the implant cannula. Finally, the software was fitted and calculated the deviation of the center of the model between the nominal and actual template model on the X, Y, and Z axis, and the Euclidean distance:

(1) $$x=\left|\left(x_{act}-x_{nom}\right)\right|,$$

(2) $$y=\left|\left(y_{act}-y_{nom}\right)\right|,$$

(3) $$z=\left|\left(z_{act}-z_{nom}\right)\right|,$$

(4) $$dxyz=\sqrt{{\left(x_{act}-x_{nom}\right)}^{2}+{\left(y_{act}-y_{nom}\right)}^{2}+{\left(z_{act}-z_{nom}\right)}^{2}}$$

Repeated measurements performed 6 times using one patient’s data as a reference value was specifically calculated. Mean deviations of angular deviation, X-coordinate deviation, Y-coordinate deviation, Z-coordinate deviation and spatial dXYZ deviation was measured. Data significance in paired groups were tested using the Tukey paired mean comparison method to evaluate whether deviations were statistically different among coordinates. Bland–Altman analysis was performed to estimate the differences between the designed template placement and actual printed template placement by using the software Origin (OriginLab, Northampton, MA, USA). Statistical analysis was performed by one-way ANOVA programming with software Origin.

## 3. Results

Accuracy Measurements

**Figure 3:**
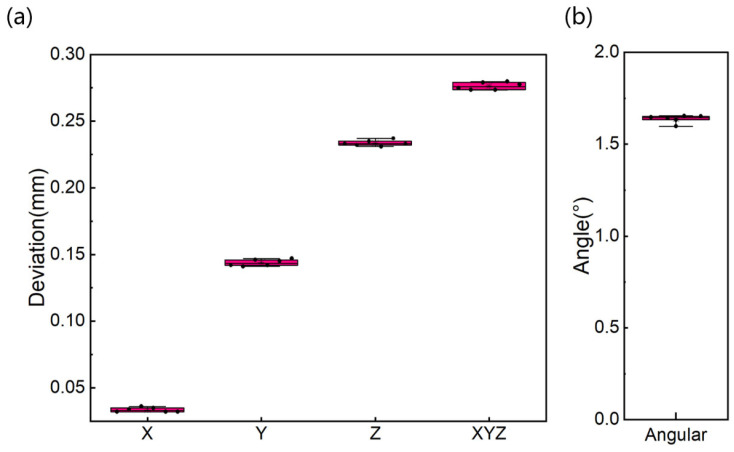
Deviation boxplots on all dimensions (**a**) and angular displacement (**b**) of the same case for 6 repeated measurements.

It takes, on average, about 20–30 min to fabricate the designed implant template. Data from different samples and 6 repeated measurements are evaluated using the aforementioned method. The results of mean, standard deviation, and range of angular accuracy of repeated measurements comparing actual and preoperative position are summarized in Table 2 and Figure 3. As shown in Figure 3, the mean ± standard deviation (SD) of the deviation on X-axis is 0.034 ± 0.002 mm (limits of the agreement range from 0.032 to 0.036 mm). The mean and SD of the deviation on Y-axis is 0.144 ± 0.002 mm (limits of the agreement range from 0.142 to 0.146 mm). The largest deviation is on the Z-axis with mean and SD of 0.234 ± 0.002 mm (limits of the agreement range from 0.232 to 0.236 mm). The mean and SD of the deviation on space Euclidean distance, dXYZ is 0.276 ± 0.003 mm (limits of the agreement range from 0.273 to 0.279 mm). The mean and SD of deviation in angle are 1.638 ± 0.021°. The values of SD and coefficient of variation are relatively small.

**Figure 4:**
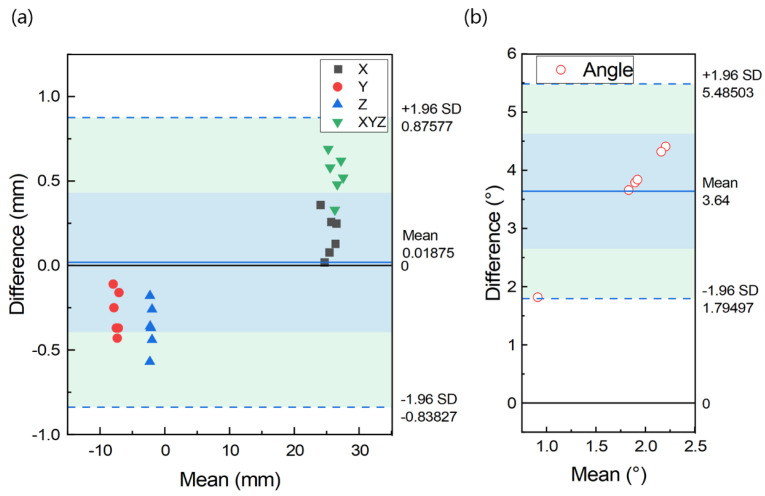
Bland-Altman plot indicating distribution of difference between designed and placed position on all dimensions (**a**) and angular (**b**) of different cases.

**Figure 5:**
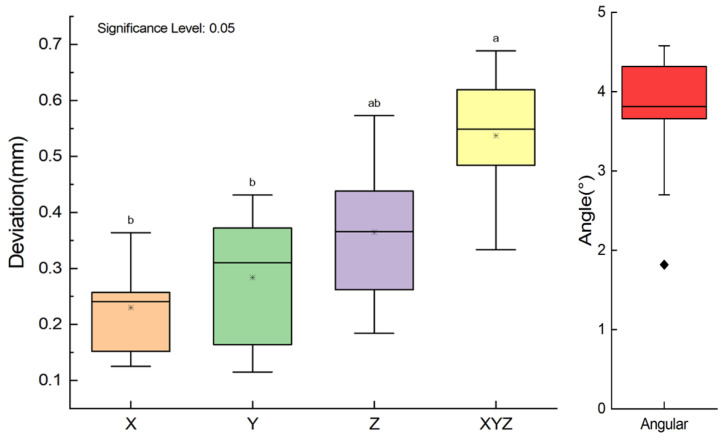
Box plot of deviation on axes (For all variables with the same letter, the difference is not statistically significant (*p* > 0.05). Different letters between groups represents significantly different (*p* < 0.05)).

Results from different samples are shown in Table 3, Figure 4 and Figure 5. In Table 3, comparison of the designed and the placed template reveals the mean ± SD of deviation on the X, Y, and Z-axis are 0.230 ± 0.085 mm, 0.284 ± 0.127 mm and 0.365 ± 0.136 mm, respectively. The mean deviation on the axes ranges from 0.230 to 0.365 mm, with maximum deviation of 0.573 mm on the Z-axis. The average deviation of the space Euclidean distance, dXYZ is 0.537 ± 0.123 mm (as shown in Table 3). The overall mean ± SD of the difference in all axes is 0.019 ± 0.857 mm (limits of agreement range from −0.838 to 0.876 mm, Figure 4). The mean ± SD of angular difference is 3.640 ± 0.941° (ranges from 1.795° to 5.485°, Figure 4). From the ANOVA analysis in Figure 5, the conclusion can be drawn that no statistically significant differences are found between the position deviation on X-, Y-, and Z-axes (*p* > 0.05).

**Figure 6:**
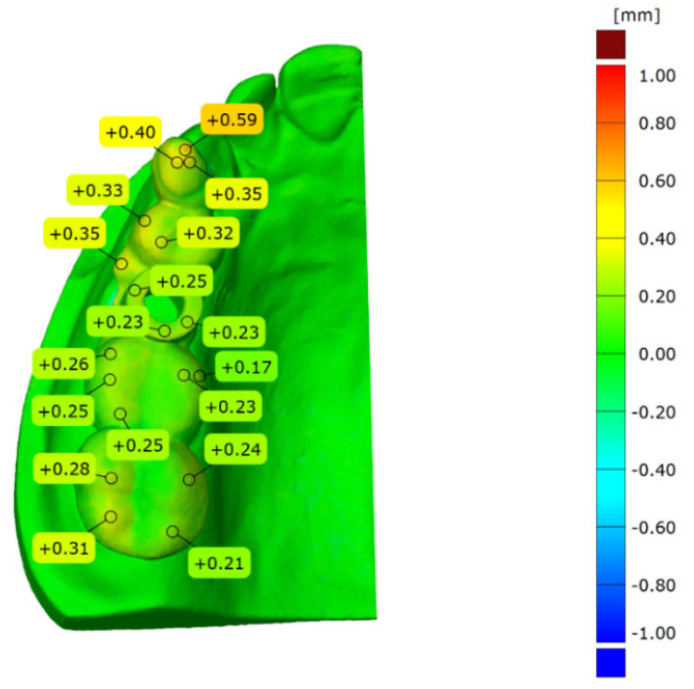
Total surface comparison between the actual placement and designed implant guide.

Total surface comparison of deviation is performed between the designed and in vitro placement model. All the models used are superimposed with the abovementioned 3-point alignment method via surface matching software GOM Inspect. The sample result is shown in Figure 6. It can be seen from Figure 6 that the model alignment is accurate and the blue color covered throughout the plaster model. The largest difference is identified on the top surface of the template guide. The deviation ranges from 0.00 to 0.59 mm.

## 4. Discussion

Surgical guides are of great importance when computer-aided implant positions are transferred to the actual clinical environment. The purpose of this research is to evaluate deviations of the printed surgical templates. Therefore, surgical templates of 20 case samples were fabricated and tested, which provides the basis for investigating the deviation between actual template placement and the planned implant position. The manufacturer of Newtom CBCT provides a measurement error of 0.004 mm for the applied measurement volume size. Both the designed and actual template are accurately superimposed, and for this step, the transformation deviation given by GOM Inspect software measures about 0.005 mm. In order to prove the repeatability of the method, two surgical templates of randomly selected cases are newly placed on the plaster model, scanned, superimposed and analyzed 6 times. The results of 6 repeated measurements show that the average standard deviations are 0.002 mm (X-axis), 0.002 mm (Y-axis) and 0.002 mm (Z-axis), 0.003 mm (dXYZ), and 0.021° (angle). These measurements display an absolute coefficient of variation of 0.009–0.053. This indicates that the errors in repositioning, optical acquisition, overlap, and geometry evaluation are very small. Therefore, the method is suitable for determining the deviation between the actual and pre-designed surgical templates in an accurate manner. Based on the above results and analysis, the conclusion can be drawn that significant differences in accuracy can be found between the coordinates, and the deviations of the placed and designed group are limited to a controllable extent.

Preoperative planning represents a vital process to successful rehabilitation of osseointegrated implantation. CBCT can reproduce bone and dental structures in a 3D model, which is superior to fan beam radiography [2]. In this study, CBCT scans were performed using the same parameter settings to obtain standard images and feature size. Guided implant surgery with the assistance of computer-aided design and manufacturing (CAD/CAM) and biomedical 3D printing for dental implant placement has been used widely in dentistry. The digital process provides excellent accuracy and can be used to predict implant position much more reliably than the conventional implant placement process applied previously [26].

A mini MEX 3D printer with length, width, and height of less than 35 cm was used in this study. The main component of the MEX 3D printer consists of three systems: motion control system, heating control system, and filament delivery system [10]. The first step in MEX process is to slice the CAD model into layers, and convert the obtained information of each layer into the path of printhead (nozzle) moving. The path information is then saved as executable machine code (G-code) and imported into the 3D printer for construction. Based on the layered information provided, the nozzle moves along the contour of the part on the XOY plane of the build platform. The nozzle will scan the contour path according to the layered information until all contour patterns of this layer are completed. Then, the nozzle rises up one layer distance to fill the contour of the next layer. Each layer printed is placed on top of the previous layer until the top layer is completed. After the top layer is printed, the construction of the part is finished [27]. Due to gravity, suspended features (>45°) in complex geometry require support to prevent collapse. Generally, it requires less than 30 min to print such a tooth supported template at 0° angle (horizon orientation).

By contrast, the stereolithography-based method requires much more time and represents a highly complex procedure [15]. Compared with the widely used stereolithography-based method, the method employed in this study to manufacture implant guides is simpler in execution and time-saving. In recent studies, Pettersson et al. [21] found that the deviation of the SLA-based template at top position of more than 100 implants measured about 0.80 mm on average, and the angular deviation was 2.26°. Cunha et al. [2] analyzed 61 implants guided by prototyped surgical guides. They found angular deviation was 2.04°, coronal, central, and apical linear deviations were 0.68 mm, 0.72 mm, and 0.82 mm, respectively. The results revealed that there was no statistically significant difference between planned and inserted positions. Yeung et al. [28] evaluated the accuracy and precision of three different implant systems. They discovered that different guided implant surgery systems contained strengths and weaknesses as revealed in the dimensional and angulation implant displacements. Yao et al. [23] compared implant guides fabricated by MEX and light-curing methods for twenty mandibular resin models with missing teeth 36 and 46. They concluded that the accuracy of surgical guide for implant placement fabricated by MEX was equally accurate to that of light-curing based guides. In our study, a similar accuracy was obtained to that of previous research. Deviation sources can be ascribed to software errors [29] or to the selected materials [25]. Due to the limitation of the manufacturing process and the applicability of the production of the implant template, in this study, the accuracy errors of the templates resulted from production and implantation mainly arising from the following aspects [30]: (1) CBCT scan and model reconstruction [31]; (2) format conversion of reconstructed models [30,32]; (3) MEX-based 3D printing process of the templates [33,34]; (4) the template placement [3,4,8,9,26,31,35,36].

In material extrusion-based 3D printing of surgical templates, the main error occurs in CAD model conversion, model slice and material shrinkage. In the CAD model conversion process, the surface of the CAD model produced by model design software is transformed to a finite number of triangular facets that approximate the surface profile of the CAD model. When the surface of the model is planar, general errors will not be generated in this process. In application, however, there is a large number of complex surfaces in the model, which cause the surface to be approximated by triangular facets. Regardless of how finely divided, there will be errors. In the model slice process, the 3D model must be discretized to 2D cross-section contour in its building direction. The discretized model can then be used for MEX-based manufacturing. This process is called slicing, and the thickness of the slice is critical and can directly affect part accuracy. For STL files processed in layers, the thickness is usually between 0.1 and 0.3 mm. Hence, the slicing process will cause damage to the continuous surface. Thus, the contours between adjacent layers in the building direction cannot overlap, otherwise it will result in errors in the shape and size of the surface, known as a “step error”. In the printing process, high-temperature printing causes printed parts to vary from the designed model in size after cooling due to shrinkage of the printing filament material. In order to avoid the influence of material shrinkage, it is necessary to consider the shrinkage factor in the design process of the 3D model, and pre-calculate and compensate for the shrinkage of the model. In addition, the shrinkage rate varies among different materials, and the shrinkage rate of the same material is also different due to different application environments, such as the shape and size of the model, and different printing equipment. In this way, the actual shrinkage rate of each material needs to be determined according to the application scenario.

In the process of forming the guide template, each step from the CBCT scan to the fabrication of the templates causes deviations. The errors gradually accumulate throughout the process [32,37], which is complicated and inevitable. For these issues, further research is in need. During manufacture and handling, special attention should be paid to improve the accuracy of templates [38]. Pre-implantation surgery planning is equally important, or even more important, than the implantation process. Based on the abovementioned error source, in this study, a 0.3 mm nozzle was equipped to the printer, layer thickness was set to 0.1 mm to obtain preferable accuracy of the printed guide template. The print temperature was set to 200 °C according to the standardized temperature for printing small parts.

An efficient way to fabricate implant templates from the chairside and a convenient method of predicting the accuracy of the MEX-printed template placement are proposed in this study, although bearing its own limitations similar to that of the previous work by [39]. The first limitation is that the placement of dental guides and casts cannot fully simulate the clinical situation in the same manner as when it is fixated into the patient’s mouth, for there may be micro-movements of natural teeth and soft tissue in the mouth. The second is that the soft tissue is not included in the dental cast used in this work, which means that the deviations may be caused as a result of missing soft tissue support of the implant guide in the bottom section. The third is that the implant system may differ from other studies or clinical situations, which can only provide a reference of an overview of the teeth-supported implant system. In addition to the limitations listed above, another is that the deviations in all coordinates were analyzed separately and were supposed to be independent, whilst there may be an inner relationship among the deviations that were not analyzed in this study. Future research should be performed on the optimization of the MEX process parameter to obtain preferable accuracy. In addition, the analysis of in vivo deviations for different oral positions with consideration of the inherent relationship should be included.

## 5. Conclusions

Based on the research results of this in vitro study, the following conclusions can be drawn: (1) Surgical guides fabricated by MEX bear relatively high accuracy for tooth supported implantation with promoted efficiency. (2) The deviation of the angle measures approximately 3.640 ± 0.941°. For position deviation, the maximum deviation direction is in the Z axis with a mean deviation of 0.365 ± 0.136 mm. (3) The mean deviation of space Euclidean distance dXYZ is 0.537 ± 0.123 mm. Hence, a method for predicting implant accuracy is proposed, which is of acceptable feasibility and reliability.

## Figures and Tables

**Table 1:** Printing parameters of the MEX process.

Quality			Fill		Speed and Temperature		Material	
Build Volume (mm)	Layer Thickness (mm)	Nozzle Size (mm)	Bottom/Top Thickness (mm)	Fill Density (%)	Speed (mm/s)	Print Temperature (°C)	Filament Type	Filament Diameter (mm)
120 × 120 × 100	0.1	0.3	0.8	100	30	200	PLA	1.75

**Table 2:** Mean, SD (standard deviation), CV (coefficient of variation) in all dimensions and angular accuracy of 6 repeated measurements from the same case.

	Min	Max	Mean	SD	CV
X (vestibule-oral, mm)	0.032	0.036	0.034	0.002	0.053
Y (mesio-distal, mm)	0.141	0.147	0.144	0.002	0.017
Z (cranio-caudal, mm)	0.231	0.237	0.234	0.002	0.009
dXYZ (mm)	0.273	0.280	0.276	0.003	0.010
Angle (°)	1.598	1.655	1.638	0.021	0.013

**Table 3:** Results of Mean, SD in all dimensions and angular data from 20 implants.

	Min	Max	Mean	SD
X (vestibule-oral, mm)	0.125	0.364	0.230	0.085
Y (mesio-distal, mm)	0.115	0.431	0.284	0.127
Z (cranio-caudal, mm)	0.184	0.573	0.365	0.136
dXYZ (mm)	0.333	0.689	0.537	0.123
Angle (°)	1.820	4.410	3.640	0.941

## Data Availability

The data presented in this study are available on request from the corresponding author.

## References

[1] Venskutonis T., Plotino G., Juodzbalys G., Mickevieiene L. (2014). The importance of cone-beam computed tomography in the management of endodontic problems: A review of the literature. J. Endodont..

[2] Cunha R.M., Souza F.A., Hadad H., Poli P.P., Maiorana C., Carvalho P. (2021). Accuracy evaluation of computer-guided implant surgery associated with prototyped surgical guides. J. Prosthet. Dent..

[3] Sanna A.M., Molly L., van Steenberghe D. (2007). Immediately loaded cad-cam manufactured fixed complete dentures using flapless implant placement procedures: A cohort study of consecutive patients. J. Prosthet. Dent..

[4] Vasak C., Strbac G.D., Huber C.D., Lettner S., Gahleitner A., Zechner W. (2015). Evaluation of three different validation procedures regarding the accuracy of template-guided implant placement: An in vitro study. Clin. Implant Dent. Relat. Res..

[5] Matta R., Bergauer B., Adler W., Wichmann M., Nickenig H. (2017). The impact of the fabrication method on the three-dimensional accuracy of an implant surgery template. J. Cranio Maxill. Surg..

[6] Herschdorfer L., Negreiros W.M., Gallucci G.O., Hamilton A. (2021). Comparison of the accuracy of implants placed with cad-cam surgical templates manufactured with various 3d printers: An in vitro study. J. Prosthet. Dent..

[7] Assche N.V., Steenberghe D.V., Guerrero M.E., Hirsch E., Jacobs R. (2007). Accuracy of implant placement based on pre-surgical planning of three-dimensional cone-beam images: A pilot study. J. Clin. Periodontol..

[8] Fang Y., An X., Jeong S.M., Choi B.H. (2019). Accuracy of computer-guided implant placement in anterior regions. J. Prosthet. Dent..

[9] Bover-Ramos F., Vina-Almunia J., Cervera-Ballester J., Penarrocha-Diago M., Garcia-Mira B. (2018). Accuracy of implant placement with computer-guided surgery: A systematic review and meta-analysis comparing cadaver, clinical, and in vitro studies. Int. J. Oral Maxillofac. Implant..

[10] Wagner M.A., Hadian A., Sebastian T., Clemens F., Schweizer T., Rodriguez-Arbaizar M., Carreño-Morelli E., Spolenak R. (2021). Fused filament fabrication of stainless steel structures—From binder development to sintered properties. Addit. Manuf..

[11] Wang H., Masood S., Iovenitti P., Harvey E.C. (2001). Application of Fused Deposition Modeling Rapid Prototyping System to the Development of Microchannels.

[12] Singh S., Singh G., Prakash C., Ramakrishna S. (2020). Current status and future directions of fused filament fabrication. J. Manuf. Process..

[13] Zhang B., Wang L., Song P., Pei X., Sun H., Wu L., Zhou C., Wang K., Fan Y., Zhang X. (2021). 3d printed bone tissue regenerative pla/ha scaffolds with comprehensive performance optimizations. Mater. Des..

[14] Baro M., Sánchez E., Delgado A., Perera A., Évora C. (2002). In vitro-in vivo characterization of gentamicin bone implants. J. Control. Release.

[15] Pugliese R., Beltrami B., Regondi S., Lunetta C. (2021). Polymeric biomaterials for 3d printing in medicine: An overview. Ann. 3D Print. Med..

[16] Narayanan G., Vernekar V.N., Kuyinu E.L., Laurencin C.T. (2016). Poly (lactic acid)-based biomaterials for orthopaedic regenerative engineering. Adv. Drug Deliv. Rev..

[17] Serra T., Planell J.A., Navarro M. (2013). High-resolution pla-based composite scaffolds via 3-d printing technology. Acta Biomater..

[18] Afonso J.A., Alves J.L., Caldas G., Gouveia B.P., Santana L., Belinha J. (2021). Influence of 3d printing process parameters on the mechanical properties and mass of pla parts and predictive models. Rapid Prototyp. J..

[19] Mishra P.K., Ponnusamy S., Nallamilli M.S.R. (2021). The influence of process parameters on the impact resistance of 3d printed pla specimens under water-absorption and heat-treated conditions. Rapid Prototyp. J..

[20] von Windheim N., Collinson D.W., Lau T., Brinson L.C., Gall K. (2021). The influence of porosity, crystallinity and interlayer adhesion on the tensile strength of 3d printed polylactic acid (pla). Rapid Prototyp. J..

[21] Pettersson A., Komiyama A., Hultin M., Näsström K., Klinge B. (2012). Accuracy of virtually planned and template guided implant surgery on edentate patients. Clin. Implant. Dent. Relat. Res.

[22] Zimmermann M., Ender A., Mehl A. (2020). Local accuracy of actual intraoral scanning systems for single-tooth preparations in vitro. J. Am. Dent. Assoc..

[23] Sun Y., Ding Q., Tang L., Zhang L., Sun Y., Xie Q. (2019). Accuracy of a chairside fused deposition modeling 3d-printed single-tooth surgical template for implant placement: An in vitro comparison with a light cured template. J. Cranio Maxill. Surg..

[24] Widmann G., Berggren J.P.M., Fischer B., Pichler-Dennhardt A.R., Schullian P., Bale R., Puelacher W. (2015). Accuracy of image-fusion stereolithographic guides: Mapping ct data with three-dimensional optical surface scanning. Clin. Implant. Dent. Relat. Res.

[25] Molinero-Mourelle P., Canals S., Gómez-Polo M., Solá-Ruiz M.F., Highsmith J.R., Viñuela A.C. (2018). Polylactic acid as a material for three-dimensional printing of provisional restorations. Int. J. Prosthodont..

[26] Hultin M., Svensson K.G., Trulsson M. (2012). Clinical advantages of computer-guided implant placement: A systematic review. Clin. Oral Implan. Res..

[27] Sharafi S., Santare M.H., Gerdes J., Advani S.G. (2021). A review of factors that influence the fracture toughness of extrusion-based additively manufactured polymer and polymer composites. Addit. Manuf..

[28] Yeung M., Abdulmajeed A., Carrico C.K., Deeb G.R., Bencharit S. (2020). Accuracy and precision of 3d-printed implant surgical guides with different implant systems: An in vitro study. J. Prosthet. Dent..

[29] Baruffaldi A., Poli P.P., Baruffaldi A., Giberti L., Pigozzo M., Maiorana C. (2016). Computer-aided flapless implant surgery and immediate loading. A technical note. Oral Maxillofac. Surg..

[30] Neumeister A., Schulz L., Glodecki C. (2017). Investigations on the accuracy of 3d-printed drill guides for dental implantology. Int. J. Comput. Dent..

[31] Tan P.L., Layton D.M., Wise S.L. (2018). In vitro comparison of guided versus freehand implant placement: Use of a new combined trios surface scanning, implant studio, cbct, and stereolithographic virtually planned and guided technique. Int. J. Prosthodont..

[32] George E., Liacouras P., Rybicki F.J., Mitsouras D. (2017). Measuring and establishing the accuracy and reproducibility of 3d printed medical models. Radiographics.

[33] Chen H., Yang X., Chen L., Wang Y., Sun Y. (2016). Application of fdm three-dimensional printing technology in the digital manufacture of custom edentulous mandible trays. Sci. Rep.-UK.

[34] El-Katatny I., Masood S.H., Morsi Y.S. (2010). Error analysis of fdm fabricated medical replicas. Rapid Prototyp. J..

[35] Assche N.V., Vercruyssen M., Coucke W., Teughels W., Jacobs R., Quirynen M. (2012). Accuracy of computer-aided implant placement. Clin. Oral Implan. Res..

[36] Cushen S.E., Turkyilmaz I. (2013). Turkyilmaz, Impact of operator experience on the accuracy of implant placement with stereolithographic surgical templates: An in vitro study. J. Prosthet. Dent..

[37] Szymor P., Kozakiewicz M., Olszewski R. (2016). Accuracy of open-source software segmentation and paper-based printed three-dimensional models. J. Cranio-Maxillo-Facial Surg..

[38] Kim T., Lee S., Kim G.B., Hong D., Kwon J., Park J.-W., Kim N. (2020). Accuracy of a simplified 3d-printed implant surgical guide. J. Prosthet. Dent..

[39] Deeb G.R., Allen R.K., Hall V.P., Whitley D., Laskin D.M., Bencharit S. (2017). How accurate are implant surgical guides produced with desktop stereolithographic 3-dimentional printers?. J. Oral Maxillofac. Surg..

